# Hypoxia driven by Caribbean Sargassum accumulation events

**DOI:** 10.1098/rsos.250322

**Published:** 2025-08-06

**Authors:** Jose Martinez Ortiz, Jenniffer Perez Perez, Roy A. Armstrong, Juan J. Cruz Motta, Travis A. Courtney

**Affiliations:** ^1^ Department of Marine Sciences, University of Puerto Rico Mayagüez, Mayagüez, Puerto Rico

**Keywords:** hypoxia, Sargassum accumulation, dissolved oxygen, biochemical oxygen demand, Sargassum brown tide, Sargassum, Caribbean

## Abstract

Sargassum accumulation events threaten coastal ecosystems across the Caribbean and have been associated with severe hypoxia. However, our understanding of Sargassum-induced hypoxia is limited by the lack of continuous monitoring of seawater dissolved oxygen during Sargassum accumulation events and the absence of decaying Sargassum oxygen uptake rates. Here, we combined time series of dissolved oxygen with remote sensing of Sargassum areal coverage in early summer and late winter at Isla Magueyes, Puerto Rico. While mild/moderate hypoxia was more frequent in summer than in winter, severe hypoxia was only observed following two distinct Sargassum accumulation events in early and mid-September 2023. We conducted incubation experiments with fresh decaying Sargassum to quantify mean ± s.e. oxygen demand rates of 0.0038 ± 0.0005 mg DO d^−1^ mg Sargassum^−1^ and incorporated these rates into a box model to show that modelled night-time was more severe with increasing Sargassum biomass and that less biomass was required to reach severe hypoxia under warmer and longer residence time scenarios. Our results demonstrate that Sargassum accumulation can drive local hypoxia and that the frequency and severity of Sargassum-induced hypoxia events in the Caribbean will likely increase under ongoing warming. These findings could be leveraged for an early warning system for future Sargassum-induced hypoxia events.

## 1. Introduction

Global oxygen content has already decreased by 2% between 1960 and 2010 with a further 1–7% decline in global oxygen values projected by 2100 due to warming and reduced ventilation to the deep ocean [1,2]. These declines can shift ocean nutrient cycles and compress marine habitats, leading to detrimental effects on global fisheries and coastal communities [2–4]. The lethal effects of deoxygenation typically emerge when aquatic dissolved oxygen (DO) concentrations fall below the hypoxia threshold of <2 mg l^−1^, but sub-lethal effects (e.g. reduced growth and reproduction, forced migration, reduced viable habitat, increased susceptibility to predation and disturbed life cycles) have been observed for moderate to weak hypoxia concentrations of up to 5 mg l^−1^ [5–8]. Consequently, ocean deoxygenation can drive declines in coastal ecosystem health and functioning with negative impacts on coastal human communities and economies that depend on these ecosystems for food and tourism [9].

DO in shallow coastal tropical ecosystems is typically more dynamic than in the open ocean and follows diel cycles with daytime photosynthesis increasing DO and night-time respiration decreasing DO [7,8,10–12]. Diel ranges in DO tend to be inversely proportional with depth (i.e. especially for depths <10 m) owing to a higher ratio of benthic biomass to water volume with additional influences by benthic community composition, hydrodynamics and organic matter inputs [3,8,13,14]. While there are comparatively few studies quantifying hypoxia in tropical systems [15], localized diel cycling modifies global mean DO with the capacity to produce localized hypoxia in tropical coastal ecosystems [7]. Consequently, weak to moderate hypoxia (<5 mg l^−1^) is already likely a common feature of coral reefs globally and is projected to increase in duration, intensity, and severity under ongoing warming [8]. Moreover, anomalous hypoxia events have been implicated in at least 20 coral reef mass mortalities globally [15] and can be driven by anomalous heat waves, eutrophication events, organic matter inputs or restricted seawater hydrodynamics [7,8].

Pelagic Sargassum accumulation events (i.e. primarily *Sargassum fluitans* and *Sargassum natans* [16]) first emerged on coastal Caribbean beaches in 2011 [17] and representing >10 million tons yr^−1^ of organic matter [18] that have been associated with localized hypoxic conditions [19–21]. Sargassum accumulation events are typically most frequent and intense during the summer months of May through August, although timing and intensity can vary between years and regions [22]. In addition to the initial Sargassum accumulation events, the subsequent decay of Sargassum causes a buildup of particulate organic material that colours the typically clear nearshore waters a murky brown colour dubbed Sargassum brown tide (Sbt) [19]. Sargassum accumulation and Sbt events can generate hypoxic conditions either through the direct consumption of oxygen by the significant increase in organic matter or through the disruption of benthic photosynthesis as both the accumulated Sargassum and Sbt can block sunlight from reaching the seafloor [19]. These Sargassum accumulation and Sbt hypoxia events can cause mortality across trophic levels and can persist for several months after initial accumulation that result in year-round impacts for selected sites [19,21,23] and can even impact human health through the production of hydrogen sulfide and ammonia gases [24].

While the links between excess organic matter inputs and hypoxia [7,15] and the co-occurrence of persistent Sargassum accumulation events and hypoxia [19,20] have previously been established, the quantitative relationship between Sargassum accumulation and Sbt events and tropical, coastal oxygen dynamics and hypoxia remains poorly constrained. This is primarily due to a general lack of continuous DO monitoring in tropical coastal systems [15] to detect discrete Sargassum accumulation-induced hypoxia events and an absence of decaying Sargassum biochemical oxygen demand rates. In this study, we addressed these shortcomings by quantifying hypoxia from autonomous DO sensors at Isla Magueyes in late summer and late winter and compared these measurements from remotely sensed Sargassum accumulation events. We then paired these field observations with laboratory incubations of fresh Sargassum to quantify the oxygen uptake rates of various amounts of decaying Sargassum. These data were then used to parametrize a generalized seawater DO box model designed to predict nightly hypoxia during Sargassum accumulation events under a range of seawater temperatures, residence times and Sargassum biomasses. This study serves as a critical first step towards developing a quantitative early warning indicator for coastal hypoxia driven by future Sargassum accumulation events to advance policy and management efforts across the Caribbean.

## 2. Methods

### 2.1. Study site

**Figure 1.**
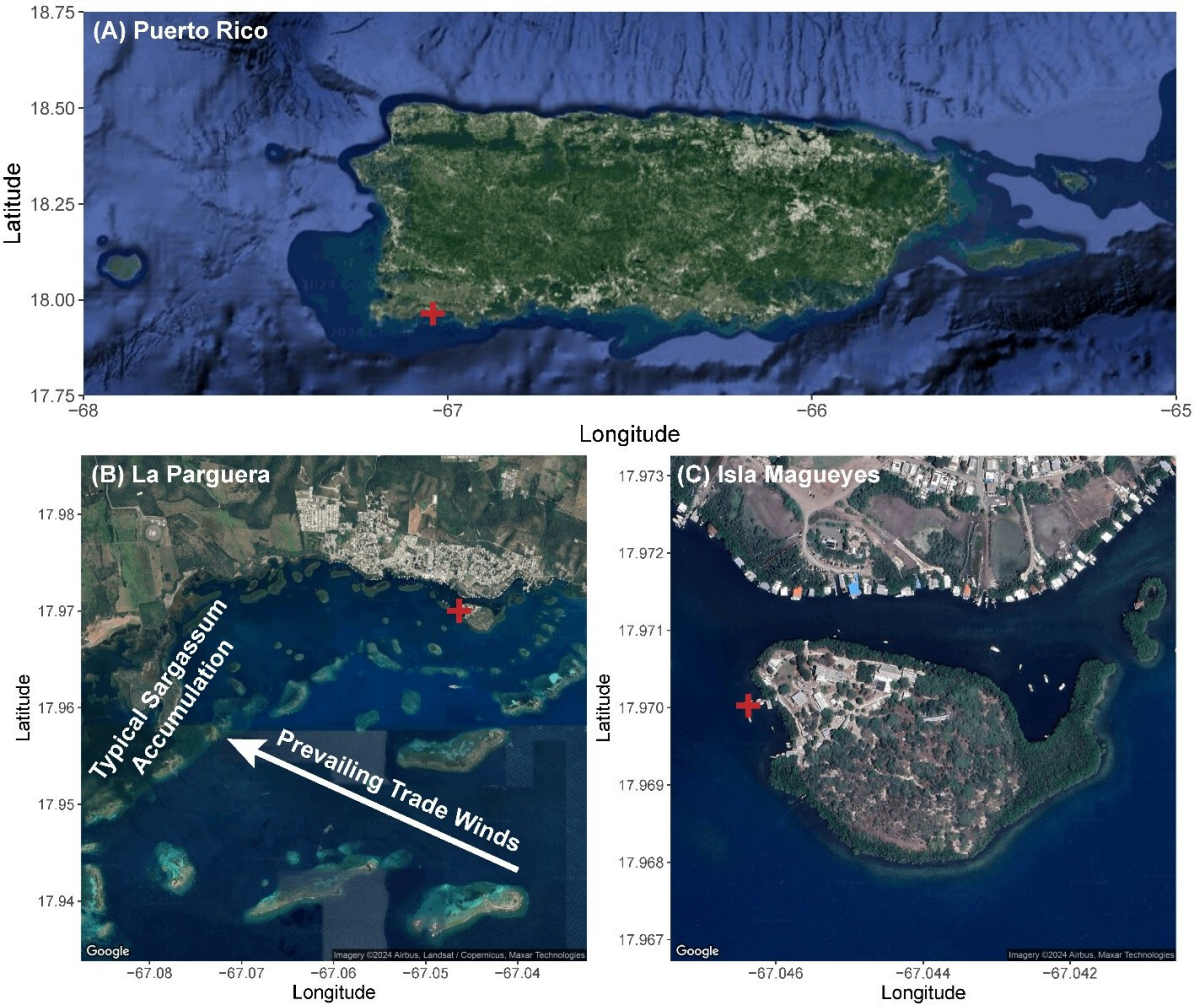
Multi-scale satellite imagery showing the location of the autonomous sensor marked by the red cross in each panel. (A) All of Puerto Rico, (B) La Parguera with the area of typical Sargassum accumulation driven by prevailing trade winds (white arrow) and (C) the Isla Magueyes study site. All images were created using Google Maps satellite data in R with the ggmap package [25].

We focused on the coastal system of La Parguera in southwest Puerto Rico, where Sargassum primarily accumulates along the southeasterly facing coasts of Isla Cueva, Isla Guayacan and La Pitahaya from April to September due to the prevailing southeasterly trade winds and westward currents (figure 1) [23,26,27]. However, shifts in the prevailing southeasterly trade winds can lead to anomalous Sargassum accumulations elsewhere in La Parguera, such as the accumulation events following southerly winds at the Isla Magueyes study site explored in this study (figure 1). We focused on the small, semi-protected embayment (2–3 m deep) on the leeward side of Isla Magueyes (figure 1), which is characterized by a mangrove-lined coastline surrounding a dense *Thalassia testudinum* seagrass bed (2–3 m depth) that is protected from the prevailing currents. Consequently, there are typically no major terrestrial organic inputs or persistent Sargassum accumulations, albeit organic matter from the mangroves and seagrass likely settles and accumulates in the benthos due to the reduced flow velocities.

### 2.2. Seawater dissolved oxygen time series

We deployed sensor packages on the seafloor at 2.3 m depth beneath the NOAA Tides and Currents Observational Platform at Isla Magueyes (figure 1) in late summer (18 August 2023 to 3 October 2023) and late winter (5 March 2024 to 9 April 2024) to autonomously record temperature (±0.1°C), conductivity (±1%), and depth (±0.5 cm H_2_O) using a Van Essen Instruments CTD-Diver, DO (±5% of the measurement or ±0.3 mg l^−1^, whichever is larger) using a PME miniDOT, and current flow speed (±3 cm s^−1^ + 3% of reading) and direction (±5° for speed >5 cm s^−1^) using a Lowell Instruments TCM-4 every 15 min. We paired these measurements with measurements of wind speed and direction and water levels above mean lower low water every 6 min by the NOAA Tides & Currents Isla Magueyes station (https://tidesandcurrents.noaa.gov/stationhome.html?id=9759110). During the summer deployment, four complete sensor packages were deployed with copper plates to protect the miniDOT sensors from biofouling, but some signs of biofouling were still observed for individual sensors (electronic supplementary material, figure S1A). To reduce the impacts of potential biofouling on the DO timeseries data, we serviced and redeployed one sensor package on 7 September 2023 and cleaned all miniDOT DO sensors on 15 September 2023. We retrieved two sensor packages on 26 September 2023 and the remaining two sensor packages on 4 October 2023. We then interpolated the four DO timeseries to a common 15 min sampling interval and reported the median of the four DO timeseries over the 18 August 2023 to 26 September 2023 period that all four sensors were deployed (electronic supplementary material, figure S1A). During the winter deployment, two complete sensor packages were deployed following the same procedures except that each of the miniDOT sensors was paired with a PME miniWiper to clean the DO sensor every 3 h to reduce potential biofouling, but again some signs of biofouling were still observed for the later days of the deployment with increased diel DO amplitude for sensor 2 relative to sensor 1 (electronic supplementary material, figure S1B). To reduce the impacts of potential biofouling on the DO timeseries data and resulting analyses, we interpolated the two timeseries to a common 15 min interval and report the mean of the two DO timeseries here over the entire period that the sensors were deployed (electronic supplementary material, figure S1B). We calculated the duration of daily hypoxia for both DO timeseries following Pezner *et al*. [8] for weak (153 µmol O_2_ kg^–1^ or approx. 5 mg l^–1^), mild (122 µmol O_2_ kg^–1^ or approx. 4 mg l^–1^), moderate (92 µmol O_2_ kg^–1^ or approx. 3 mg l^–1^), and severe (61 µmol O_2_ kg^–1^ or approx. 2 mg l^–1^) hypoxia. Duration represents the total amount of time that DO was below the respective threshold(s) for each day [8].

### 2.3. Sargassum remote sensing

Surface Sargassum was monitored using high-resolution (3 m) satellite imagery from PlanetScope Super Dove from August to September 2023 and March to April 2024. Prior to analysis, images with significant cloud coverage were excluded and a radiometric correction was performed on the remaining images using ACOLITE (https://odnature.naturalsciences.be/remsem/software-and-data/acolite) to ensure accurate water-leaving radiance measurements [28]. The Normalized Difference Vegetation Index (NDVI) was then applied using the near-infrared and red bands to differentiate Sargassum from other features, following established methods [23,26]. While detection of Sargassum in coastal waters via remote sensing represents a significant ongoing problem, the NDVI remains a strong predictor of coastal Sargassum coverage [23] and we visually assessed the study site several times a week throughout the sensor deployment to confirm whether Sargassum was indeed accumulating during periods of high NDVI values to validate remote sensing accuracy of Sargassum. The total and percent cover by Sargassum within specified areas of interest near the sensor site, around Isla Magueyes, and around La Parguera (electronic supplementary material, figure S2) were then reported for each image.

### 2.4. Sargassum biochemical oxygen demand incubations

We conducted a series of five laboratory-based biochemical oxygen demand (BOD) incubations (21 March 2024 to 10 June 2024) to quantify oxygen uptake rates for freshly decaying Sargassum following a modification of Standard Method 5120b for BOD [29] with the addition of varying amounts of freshly collected Sargassum (incubations started same day as collection) from nearshore areas (freshly stranded or floating at sea) around Puerto Rico. All Sargassum used in the incubations was fresh, non-decomposed, lacking epiphytes, and included both *Sargassum natans* and *Sargassum fluitans*. For each incubation, we filled 12 replicate 300 ml bottles with oxygenated seawater collected from the autonomous sensor station with 300 µl each of prepared phosphate buffer, MgSO_4_, CaCl_2_, and FeCl_3_. One bottle was used as a control, one bottle was used to assess method accuracy with a 1.0 ml addition of glutamic glucose acid (equivalent to 2 mg l^–1^ BOD), and the other 10 bottles received varying amounts of fresh Sargassum. There was insufficient source water collected for one of the incubation experiments resulting in 11 total bottles with 1 control, 1 glutamic glucose acid, and 9 Sargassum treatments. For each treatment, pieces of fresh Sargassum leaves were dried with a paper towel, cut using an exacto knife, weighed on a Mettler Toledo ME204T (±0.1 mg), and added to the respective bottles for a 5 day incubation period at 20 ± 3°C such that fresh Sargassum treatments ranged from 0 up to 111.1 mg (see code and data release for full table of Sargassum treatments). This range was selected based on the results of the initial incubations, wherein the final DO for concentrations exceeding 100 mg per 300 ml reached severe hypoxia (i.e. <2 mg l^−1^) after the 5 day incubation period. We therefore restricted our BOD experiments to within this range to intentionally avoid anaerobic respiration of Sargassum. These experiments therefore provide novel measurements of aerobic Sargassum decay in a controlled laboratory setting and were not meant to mimic *in situ* conditions. We calibrated an Orion AUTO-STIR BOD Probe (±0.1 mg l^–1^) using 100% water saturated air prior to each day of measurements and measured the initial and final DO of each bottle before and after incubation to calculate BOD for each sample as difference between initial and final DO. We multiplied the 5 day BOD for each bottle by 0.3 l to determine the total milligrams of DO consumed by decomposing Sargassum in the bottle and divided this number by the respective incubation duration in days to determine the mean daily DO consumption rate (mg d^–1^). We then constructed a linear mixed-effects model (LMM) of daily DO consumption rate (mg d^–1^) versus fresh Sargassum (mg) from these incubation experiments to determine the respiration rate per Sargassum biomass (mg DO d^–1^ mg Sargassum^–1^) from the slope of the model and the respiration rate of the source water collected from the sensor site (mg DO d^–1^) from the intercept of the model. We included random slopes and intercepts in the LMM for each of the five independent incubations to account for any potential differences in respiration rates by the freshly collected Sargassum (slope) or source water (intercept) between incubations.

### 2.5. Modelling Sargassum-driven hypoxia

We adapted the simple coral reef DO box model developed by Pezner *et al*. [8] to account for seawater flow rates, gross primary production, and respiration for use in R and included an additional Sargassum respiration term to generalize predictions for how much Sargassum biomass would be required to generate hypoxia across a range of potential *in situ* tropical ecosystem conditions. Briefly, the oxygen mass balance was determined for a simulated 1 m^3^ coral reef by the following equation:


$$\frac{dDO}{dt}=F_{\text{sw-i}}-F_{\text{sw-o}}+F_{PP}-F_{R}-F_{S},$$

where *F*_sw-i_ represents the flux of seawater into the box, *F*_sw-o_ represents the flux of seawater out of the box, *F*_PP_ represents gross primary production increasing from 0 at night-time to a maximum of 40 mmol O_2_ m^–2^ h^–1^ at noon before declining back to 0 over a 12 h light cycle developed by Falter *et al*. [30], *F*_R_ represents the constant respiration rate of the system (i.e. water column, benthic and sediment processes) with a base rate starting at 12 mmol O_2_ m^–2^ h^–1^ used by Pezner *et al*. [8] that changes based on temperature and oxygen solubility and *F*_S_ represents the Sargassum respiration rate in units of mmol O_2_ m^–2^ h^–1^. While the *F*_PP_ and *F*_R_ rates used here were adapted from previous studies [8,30], these rates are within the range of rates observed for tropical seagrass meadows [31]. Additionally, we assumed that air–sea gas exchange was negligible compared to other processes due to stagnant surface waters and inhibition of mixing caused by the floating mats of Sargassum, but further research is needed to evaluate oxygen fluxes into and out of decaying Sargassum mats to evaluate this assumption. We calculated *F*_S_ for a range of Sargassum biomass by multiplying the slope of the LMM converted to units of mmol O_2_ m^–2^ h^–1^ mg Sargassum^–1^ by the respective amount of Sargassum biomass considered by the model scenario. We explored residence times of 1 and 5 h (e.g. faster versus slower seawater exchange rates following [8]), seawater temperatures of 28 and 31°C (e.g. winter versus summer Caribbean seawater temperatures observed in this study) and fresh Sargassum biomass ranging from 0 to 10 kg (e.g. to exceed the full range of open ocean Sargassum biomass concentrations of up to 6 m^2^ observed by [32]). While the range of benthic metabolism, residence times, temperature, and Sargassum biomass will nonetheless vary widely across the Caribbean, these initial parameters were chosen with the range of potential conditions to explore the interaction between these drivers in a generalized scenario that could be further tuned and parametrized to match individual site parameters to improve site-specific predictions. We used the function ode [33] to numerically integrate the ordinary differential equation iteratively over 14 days for each combination of residence times, seawater temperatures, and fresh Sargassum biomass and extracted the minimum night-time DO of the last four days from the modelled DO output. The final model outputs were converted from units of µmol kg^–1^ to mg l^–1^ based on seawater density calculated using the *respR* toolbox in R [34] to report all DO in mg l^–1^ throughout the paper.

## 3. Results

### 3.1. Summer 2023 time series

**Figure 2.**
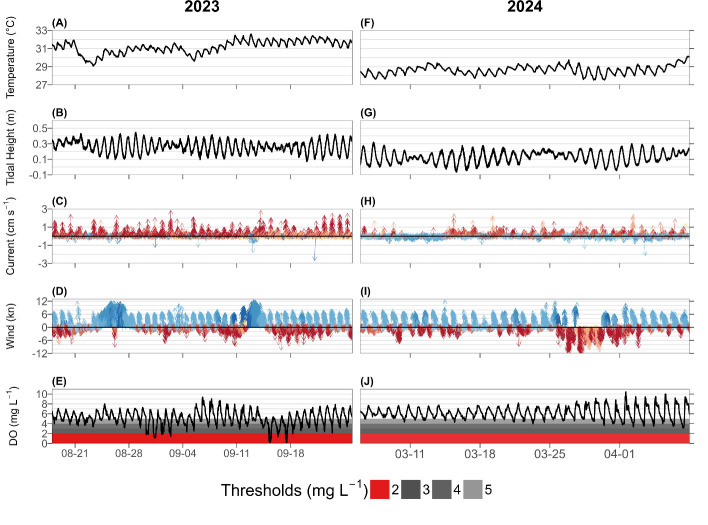
Time series of different parameters during summer 2023, including temperature (A), water level (B), current speed and direction (C), wind speed and direction (D), and dissolved oxygen (E), and during winter 2024, including temperature (F), water level (G), current (H), wind (I), and dissolved oxygen (J). Grey and red boxes indicate a different hypoxic threshold: weak hypoxia (<5 mg l^–1^) is light grey, mild hypoxia (<4 mg l^–1^) is grey, moderate hypoxia (<3 mg l^–1^) is dark grey, and severe hypoxia (<2 mg l^–1^) is in red.

Seawater temperatures ranged from approximately 29 to 33°C throughout the time series with slightly cooler temperatures at the beginning of the time series increasing throughout the sensor deployment (figure 2A). Daily minima seawater temperatures were observed in the early morning, and daily maxima were observed in the afternoon (figure 2A). Seawater flow ranged up to 3 cm s^–1^ and was primarily northward with a mean current speed of 0.50 cm s^–1^ (figure 2C). Water levels ranged from approximately 0.1 to 0.4 m above mean lower low water throughout the time series and followed a typical tidal signal with smaller tidal ranges observed during neap tide (approx. 0.2–0.4 m) in mid-August, early September, and mid-September and larger tidal ranges observed during spring tides (approx. 0.1–0.4 m) in late August, mid-September and late-September (figure 2B). Winds typically ranged up to six knots and followed a diel pattern with stronger, southeasterly winds during the afternoon and weaker northwesterly winds during the evening (figure 2D). Conversely, there were two periods of sustained approximately 12 knot southerly winds from 24 to 27 August and 9 to 14 September (see periods of sustained blue arrows in figure 2D). Seawater DO followed a typical diel pattern throughout the time series with minima observed in early morning before sunrise and maxima observed in mid-afternoon (figure 2E). However, diel DO varied in its range throughout the time series with approximately 4–7 mg l^–1^ observed from 18 to 30 August, approximately 1–7 mg l^–1^ from 30 August to 2 September, approximately 3–7 mg l^–1^ from 2 to 15 September , approximately 0–5 mg l^–1^ from 15 to 18 September and approximately 3–7 mg l^–1^ from 15 to 26 September (figure 2E).

### 3.2. Winter 2024 time series

Seawater temperatures followed a similar diel pattern with a gradual warming from approximately 28 to 30°C throughout the time series (figure 2F). Current speeds were typically <1 cm s^–1^ with a mean current speed of 0.95 cm s^–1^ that oscillated between northerly and southerly flow every few days (figure 2H). Water levels varied between approximately 0 and 0.3 m above mean lower low water with larger tidal ranges of up to approximately 0.3 m observed in early March, mid-March, and early April and smaller tidal ranges of up to approximately 0.1 m during neap tides in mid-March and late March (figure 2G). Winds typically ranged up to six knots and followed a diel pattern with stronger, southeasterly winds during the afternoon and weaker northwesterly winds during the evening (figure 2I). Conversely, there was a period of sustained approximately 12 knot northerly winds from 25 March to 1 April (figure 2I). Seawater DO followed a typical diel cycle with early morning minima and mid-afternoon maxima that varied from approximately 5 to 8 mg l^–1^ with a slight increase in diel range throughout the sensor period up to approximately 4–9 mg l^–1^ (figure 2J). Finally, there is a summary statistics table for the parameters we measured in the electronic supplementary material (table S1).

### 3.3. Sargassum and hypoxia events

**Figure 3.**
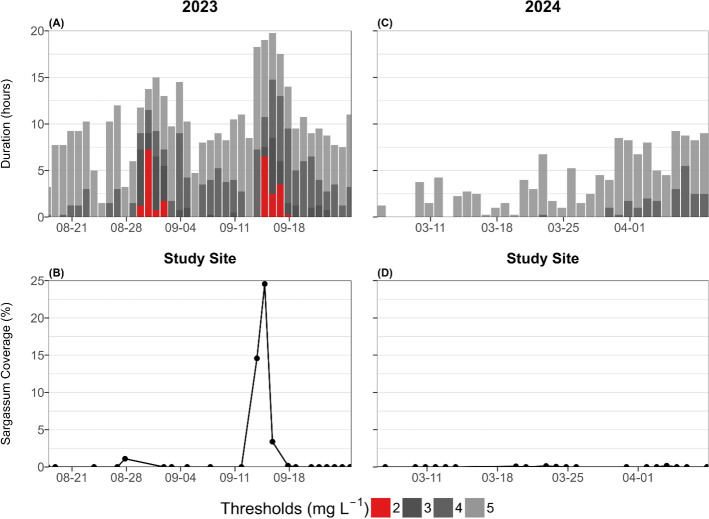
Stacked bar plot of duration of hypoxia in hours for late winter 2024 (C) and late summer 2023 (A). Grey and red bars indicate a different hypoxic threshold: weak hypoxia (<5 mg l^–1^) is light grey, mild hypoxia (<4 mg l^–1^) is grey, moderate hypoxia (<3 mg l^–1^) is dark grey, and severe hypoxia (<2 mg l^–1^) is in red. Line graph with dots representing raw data of Sargassum (NDVI) percent area coverage for late summer 2023 (B) and late winter 2024 (D) for the sensor package location.

During the late summer sensor deployment, weak hypoxia was observed for approximately 5–10 h d^–1^ every night of the DO time series data with duration increasing to approximately 10–15 h d^–1^ during the 30 August to 2 September event and approximately 15–20 h d^–1^ during the 15 September to 18 September event (figure 3A). Mild hypoxia followed a similar trend albeit with reduced duration of approximately <5 h d^–1^ increasing to approximately 8–12 h d^–1^ during the 30 August to 2 September event and approximately 10–15 h d^–1^ during the 15 September to 18 September event (figure 3A). In contrast, moderate hypoxia was observed for approximately 5–10 h d^–1^ and severe hypoxia was observed for approximately 1–6 h d^–1^ for both the 30 August to 2 September and 15 September to 18 September events with moderate hypoxia observed for 1−2 h d^–1^ following each of the events (figure 3A). During the late winter sensor deployment, weak hypoxia was observed for approximately 0–5 h d^–1^ at night for the DO time series data with duration increasing to approximately 5–10 h d^–1^ in the beginning of April (figure 3B). Only 1.5 h of mild hypoxia were observed during the entire month of March with approximately 0–5 h d^–1^ of mild hypoxia observed from 1 April to 9 April (figure 3B). There were no observations of moderate or severe hypoxia during the late winter 2024 sensor deployment.

### 3.4. Remotely sensed Sargassum coverage

**Figure 4.**
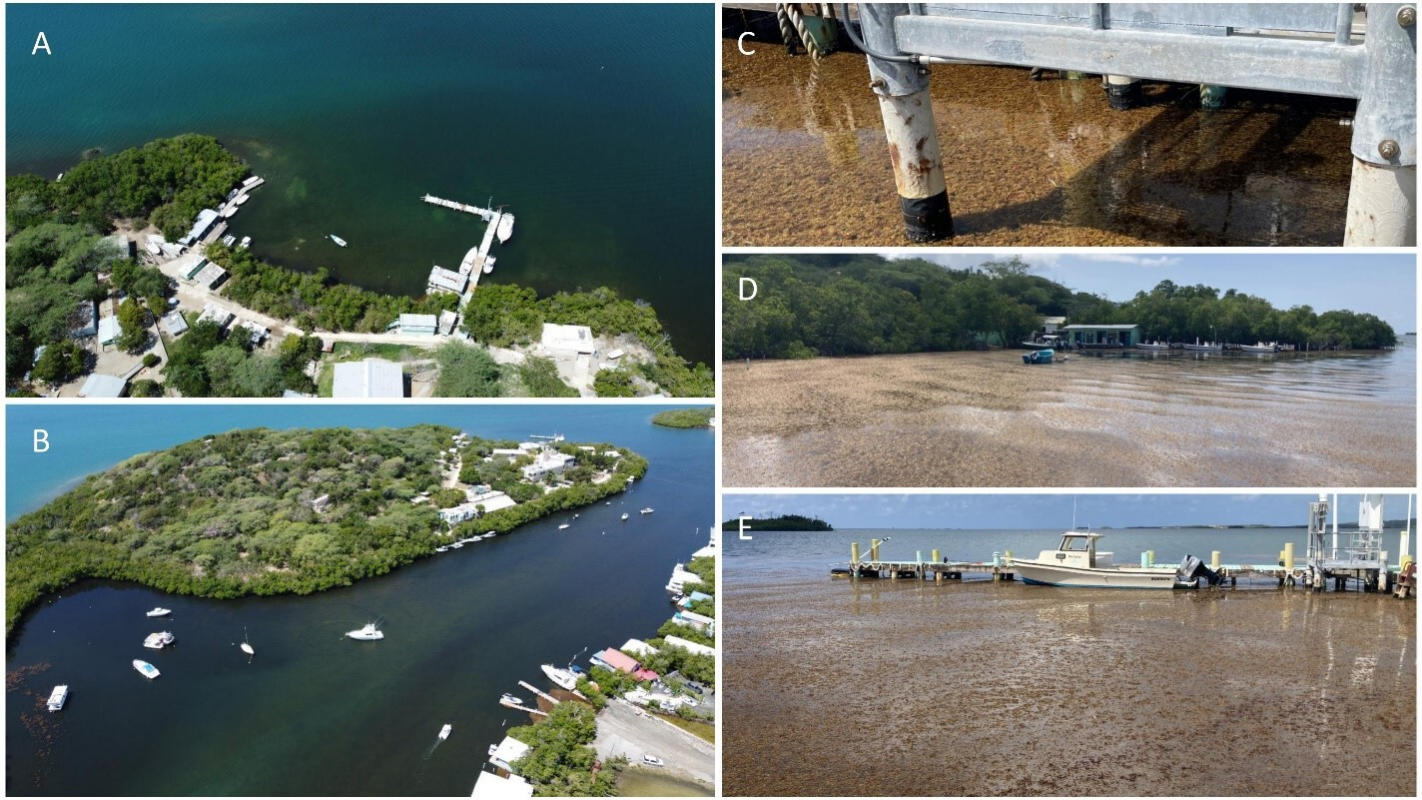
Photographs of fresh Sargassum and Sargassum brown tide around Isla Magueyes. (A) Sargassum brown tide discoloration around the study site at the Isla Magueyes dock on 1 September 2023 (photo by R.A.A.). (B) Fresh Sargassum accumulation and Sargassum brown tide in the adjacent channel of Isla Magueyes on 1 September 2023 (photo by R.A.A.). (C) Photo of fresh Sargassum accumulation covering the surface underneath the NOAA Tides and Currents Observational Platform where the sensors were deployed at Isla Magueyes on 14 September 2023 (photo by J.A.M.). (D,E) The waters surrounding the study site covered by fresh Sargassum accumulation on 14 September 2023 (photos by T.A.C.).

Throughout the late summer observation period, no Sargassum was observed for most of the time series except for two distinct Sargassum accumulation events resulting in a mean ± s.d. coverage of 2.08 ± 6.07% (figure 3B). The first event occurred on 28 August when 1.1% of the study area was covered by fresh Sargassum. This remotely sensed accumulation event was confirmed by personal observations of Sargassum accumulation and dead fish in the channel on the north side of Magueyes and by drone imagery showing minimal Sargassum accumulation and extensive delocalized Sbt primarily in the channel to the north of Isla Magueyes on 1 September (figure 4A,B). The second event started with 15% on 14 September, reached maximum coverage of 25% on 15 September and then decreased to 3% on 16 September (figure 3B). This remotely sensed accumulation event was confirmed by direct observations of extensive Sargassum accumulation at the study site on 14−15 September (figure 4C,D,E). After 16 September, only trace amounts of Sargassum were detected (0–0.17%) for the rest of the late summer observation period (figure 3B). Conversely, the late winter observational period had minimal to no Sargassum accumulation for the entirety of the observation period with mean ± s.d. coverage of 0.02 ± 0.05% observed at the study area (figure 3D). Sargassum detection from satellite imagery was not possible for 20−23, 25−26, 29−31 August, 1, 4, 6−7, 9−11, 13, 20 September, 5, 7−8, 14−18, 21, 26−29, 31 March or 6, 9 April due to excessive shadows or cloud cover.

### 3.5. Sargassum incubation experiments

**Figure 5.**
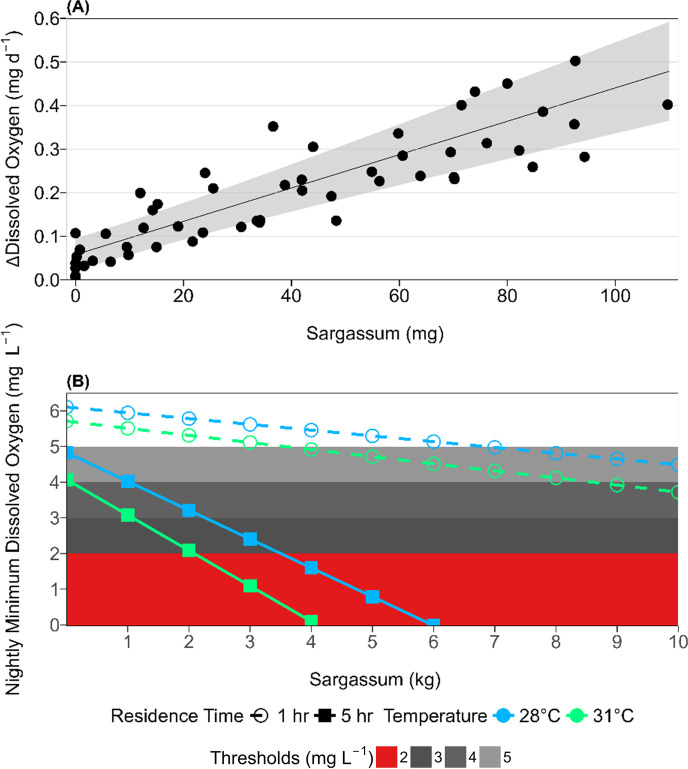
Sargassum biochemical oxygen demand rates and modelling night-time DO minima. (A) Δdissolved oxygen represents the daily DO consumption rate from the biochemical oxygen demand incubations plotted against fresh Sargassum weight (mg) from the respective incubation. Each point represents the results for a single bottle, the black line indicates the slope from the linear mixed-effects model (LMM) and the grey ribbon represents the 95% confidence interval of the slope from the LMM. (B) The nightly predicted minimum DO versus Sargassum biomass is plotted from the box model outputs. The open circles denote a 1 h residence time, filled squares represent a 5 h residence time. The dark blue line represents predicted DO at 28°C and the light blue line represents 31°C. The grey and red boxes indicate their respective hypoxia thresholds: weak hypoxia (<5 mg l^−1^) is light grey, mild hypoxia (<4 mg l^−1^) is grey, moderate hypoxia (<3 mg l^−1^) is dark grey, and severe hypoxia (<2 mg l^−1^) is in red.

Each BOD incubation met all quality assurance protocols and the mean ± standard deviation of the five glutamic glucose acid additions (+2.0 mg l^−1^ BOD) was 2.0 ± 0.3 mg l^−1^ relative to the source seawater. Fresh Sargassum biomass was positively correlated with BOD (*p* < 0.001, *t* = 7.95, d.f. = 48), resulting in a mean ± s.e. slope of 0.0038 ± 0.0005 mg DO d^−1^ mg Sargassum^−1^ and intercept of 0.06 ± 0.02 mg DO d^−1^ for the source water from the sensor location (figure 5A).

### 3.6. Modelling Sargassum-driven hypoxia

The DO predictive box model from Pezner *et al*. [8] used a base respiration rate of 12 mmol DO h^−1^ (9216 mg DO d^−1^) and including the respiration rate from the LMM adds an additional 4.95 mmol DO h^−1^ kg Sargassum^−1^ (3800 mg DO d^−1^ kg Sargassum^−1^). The 1 h residence time at 28°C simulation predicted weak hypoxia when Sargassum exceeded approximately 7 kg (figure 5B). Similarly, the 1 h residence time at 31°C simulation predicted weak hypoxia when Sargassum exceeded approximately 3.5 kg and mild hypoxia when Sargassum exceeded approximately 8.5 kg (figure 5B). The 5 h residence time at 28°C simulation predicted weak hypoxia at approximately 0 kg Sargassum, mild hypoxia at approximately 1 kg Sargassum, moderate hypoxia at approximately 2 kg Sargassum, and severe hypoxia at approximately 3.5 kg Sargassum (figure 5B). Similarly, the 5 h residence time at 31°C predicted mild hypoxia at approximately 0 kg Sargassum, moderate hypoxia at approximately 1 kg Sargassum, and severe hypoxia at approximately 2 kg Sargassum (figure 5B).

## 4. Discussion

In this study, we identified a direct relationship between Sargassum accumulation events and severe hypoxia events at the Isla Magueyes study site (figures 2 and 3). During the DO and Sargassum accumulation monitoring periods in late summer and late winter, just two distinct severe hypoxia events were observed 1−2 days after anomalous accumulations of Sargassum at the study site in late summer (figures 2E and 3A). This timing agrees with previous studies documenting hydrogen sulfide gas production (sulfate reduction) after 48 h of Sargassum accumulating on beaches [24]. While mild to moderate hypoxia was more frequently observed during the warmer summer months, no severe hypoxia was observed in the absence of Sargassum (figure 2E,J). This strongly implicates Sargassum accumulation as the most probable driver of these severe hypoxia events (figure 3B,D). Additionally, we tested the causal driver of Sargassum-driven hypoxia events through the Sargassum 5 day BOD incubation experiments and found that DO consumption increased linearly with Sargassum biomass at a rate of 0.0038 ± 0.0005 mg DO d^−1^ mg Sargassum^−1^ (figure 5A; but see subsequent discussion of reduced DO consumption rates as DO becomes limiting). However, at high concentrations of Sargassum biomass, we expect the linear relationship to plateau as respiration shifts to energy processes dominated by anaerobic pathways. While this rate was slightly lower than the approximately 0.0096–0.011 mg DO d^−1^ mg Sargassum^−1^ respiration rate reported by Smith *et al*. [35], that study reported respiration over 4 h by living Sargassum compared to the 5 day Sargassum decay rate reported here (figure 5A). We applied our BOD rate to varying concentrations of Sargassum in the box model and found that nightly minimum DO decreased with increasing Sargassum (figure 5B). These findings corroborate point measurements of coastal hypoxia in areas of persistent Sargassum accumulation in La Parguera and Mexico [27,19,21,36] by establishing that severe hypoxia events at the study site only coincided with Sargassum accumulation events (figures 2 and 3) and that hypoxia severity scaled with Sargassum coverage in the field (figure 3) and Sargassum biomass in the incubations and model (figure 5).

While persistent Sargassum accumulation typically occurs on the western coastlines of La Parguera (figure 1B) [23,26,27], this study leveraged novel Sargassum accumulations to explore the relationship between Sargassum and coastal hypoxia. Both hypoxia events in this study (figure 3) directly followed extended periods of sustained southerly winds from 24 to 27 August and 9 to 14 September (figure 2E) associated with the nearby passage of Hurricane Franklin (first event) and Hurricane Lee (second event). While direct Sargassum accumulation was lower at the study site during the first event (figure 3B), there was more extensive accumulation in the nearby channel with a decentralized Sbt extending to the study site (figure 4A,B). Moreover, while a localized fish kill was observed in the channel, no mortality was observed at the study site. Nonetheless, night-time DO decreased at our study site from approximately 4 mg l^−1^ during 18−30 August to approximately 2 mg l^−1^ (minimum = 1.16 mg l^−1^) during the 30 August–2 September hypoxia event (figure 2E and 3A). This suggests the first hypoxia event at the sensor site was primarily a de-localized event with more extreme hypoxia and fish mortality occurring in the channel and less intensely impacting the sensor location through the combined effects of Sargassum accumulation and Sbt at the sensor site. During the second event, there was a significant accumulation of fresh Sargassum at the study site, peaking at 25% coverage on 15 September (figures 3B and 4C,D,E). Similar to the first event, we observed a decline in night-time DO at our study site, but it was more intense during this event, decreasing from approximately 3 mg l^−1^ during 2−15 September to approximately 0 mg l^−1^ (minimum = 0.06 mg l^−1^) on 15 and 17 September (figure 2E and 3A). While reduced wind speeds, currents, and tidal ranges can contribute to hypoxia events [15], severe hypoxia was observed during typical wind speeds and current velocities, proceeded neap tides during the first event, lagged neap tides during the second event, and was not associated with neap tides at the start of the summer timeseries (figure 2). While these factors may have modified the intensity of the events, severe hypoxia was only observed following the two distinct Sargassum accumulation events (figure 3), suggesting Sargassum accumulation was the primary driver of severe hypoxia in this study.

Climate change is expected to increase the frequency and intensity of hypoxia on tropical coral reef and seagrass ecosystems [8] primarily due to the decreasing solubility of oxygen in seawater and increasing metabolic rates under ongoing warming [2,8]. Thus, as warming continues, marine organisms consume more oxygen in a warmer environment that contains less oxygen [2,8]. The effect of warming on hypoxia was reflected by the increased frequency of night-time moderate hypoxia (excluding hypoxia events) in late summer with no recorded moderate or severe hypoxia events in late winter (figures 2 and 3). This agrees with previous assessments that warming will increase the frequency, intensity, and severity of hypoxia events on coral reef ecosystems [8]. Moreover, these events can be exacerbated by reduced wind speeds that inhibit air–sea gas exchange and reduced currents and tidal ranges that replenish sites with oxygenated waters from elsewhere [15]. Thus, the protection from the prevailing wind directions (figure 1) and reduced current speeds (figure 2) at Isla Magueyes may have exacerbated nightly weak to mild summertime hypoxia at the study site. This nightly weak to mild hypoxia lowers the threshold for Sargassum biomass required to reach hypoxia and highlights the important interactions between warming and organic matter loading for triggering hypoxia events (*sensu* [15]). Sargassum accumulation is typically most intense during the summer months [22] and therefore interacts with warmer waters to increase the frequency and intensity of summertime hypoxia events. As a result, assuming Sargassum accumulation events continue (e.g. [37]), Sargassum-driven hypoxia events are therefore likely to increase in frequency, intensity, and spatial coverage under ongoing ocean warming.

We further explored this interaction of seawater hydrodynamics, warming, and Sargassum biomass in our box model to better understand and predict Sargassum hypoxia events under warming and different residence times (figure 5). Night-time DO decreased with increasing Sargassum biomass under every simulation of residence time and temperature (figure 5B). Scenarios with longer residence times had lower night-time DO compared to scenarios with shorter residence time scenarios for the same biomass of Sargassum (figure 5B). Similarly, night-time hypoxia was more extreme for the 31°C scenario compared to the corresponding 28°C scenario with the same residence time (figure 5B). However, there was an interaction between residence time, warming, and Sargassum biomass with substantially less Sargassum required to reach severe hypoxia for the warmer, longer residence time scenario. For example, while the difference between the 1 h residence time scenarios increased slightly from approximately 0.4 mg l^−1^ at 0 kg of Sargassum to approximately 0.8 mg l^−1^ at 10 kg of Sargassum, the difference between the 5 h residence time scenarios increased from approximately 0.8 mg l^−1^ at 0 kg Sargassum to approximately 1.5 mg l^−1^ at 4 kg Sargassum (figure 5B). The potential transport of Sargassum (not included in the model) complicates the relationship with residence time explored here since shorter residence times would be more likely to export Sargassum out of the system (less severe hypoxia in the real world compared to model), while longer residence times would increase retention of Sargassum in the system (resulting in similar hypoxia to modelled results). In addition to export, the decomposition of Sargassum biomass itself would decrease the total amount of biomass available for decomposition resulting in reduced BOD and a return to ‘normoxia’ similar to our observations of distinct hypoxia events at Isla Magueyes (figure 2). We assumed that air–sea gas exchange was negligible owing to the inhibition by floating Sargassum, but further research would be needed to test the degree to which this assumption biased our results towards extreme hypoxia. We also based our modelling results on the BOD rates from the incubations in this study, which never reached anoxia (minimum DO = 0.20 mg l^−1^). We would expect BOD rates to decrease under increasingly hypoxic oxygen conditions as Sargassum decomposition transitions from aerobic respiration to less efficient organic matter respiration pathways such as the sulfate reduction observed on beaches [24]. Reduced rates of Sargassum organic matter respiration in low oxygen conditions could in part explain the persistent, year-round Sargassum accumulations observed elsewhere [19,23,26], and future studies should explore longer-term sustained Sargassum degradation to test this hypothesis.

Although we observed no significant mortality of seagrass or other organisms at the sensor site, organisms at the study site were exposed to potentially lethal thresholds of hypoxia a second time in less than a month and were chronically exposed to sub-lethal hypoxia for the entirety of the late summer study period (figures 3 and 5). While severe hypoxia can induce mortality, tolerance thresholds vary significantly between species and many organisms can persist through hypoxic events by behavioural avoidance, decreased metabolisms, or physiological adaptation [6]. For example, organisms at the study site are likely pre-adapted to the consistent summertime weak/mild night-time hypoxia (figure 2), which may explain the lack of detectable mortality during the Sargassum-induced hypoxia events at the study site. Further research should be conducted to explore the lethal and sub-lethal DO thresholds for tropical coastal organisms [6–8,15,38] to better understand and project the impacts of future Sargassum-induced hypoxia events. The impacts of short-term, smaller spatial scale events in this study contrast other areas of persistent Sargassum accumulation in La Parguera and Mexico [19,23,26]. At these locations, Sargassum accumulation events can be more frequent than the recovery time of keystone species and can even persist year-round, resulting in phase shifts in coastal marine habitats away from mangroves and seagrasses [19,26]. As a result, Sargassum accumulation events are accelerating declines in coastal Caribbean ecosystem functions and services such as essential fisheries nursery habitat, shoreline protection, and carbon sequestration that mangroves and seagrass systems provide [39,40].

### 4.1. Recommendations

Here, we have shown that Sargassum accumulation can drive local hypoxia and is likely increasing under ongoing warming, resulting in a need to work towards increased prediction to help with future marine planning to be better equipped to deal with these effects. We suggest that if the biomass of incoming Sargassum is known via remote sensing techniques [32], models such as the one presented here can be further developed and adapted for individual site characteristics such as depth, temperature, and residence time to estimate whether severe hypoxia is likely for a given influx of Sargassum biomass. Remote sensing of Sargassum biomass (e.g. [32]) coupled with simple models such as the one presented here could be further developed to generate potential early warning systems for Sargassum hypoxia events in the future and could be useful for the Caribbean communities currently assessing coastal vulnerability to Sargassum inundation [18] and developing policy-related decisions on how to deal with Sargassum inundations [41]. This study therefore presents critical first steps in developing an early system for Sargassum hypoxia events by directly implicating Sargassum accumulation in coastal hypoxia, quantifying the biological oxygen demand of decaying Sargassum, and modelling how residence time, warming, and Sargassum biomass interact to generate hypoxia events. Regardless, coastal hypoxia and Sargassum accumulation events are both expected to continue to intensify under climate change, suggesting that reduced greenhouse gas emissions and localized organic matter inputs are key to reduce the frequency, intensity, and severity of coastal hypoxia events in the Caribbean.

## Data Availability

The data and code that support the findings of this study are openly available in a GitHub repository. The repository contains all relevant datasets, scripts, and necessary files to reproduce the results shown in this work. The repository can be accessed at https://github.com/jmartinez2k/HypOxSarg/tree/main and data and code have been archived within the Zenodo repository [42].
